# Trends in mortality and hospitalisations for cardiovascular, kidney and liver disease in people with type 2 diabetes in England, 2009–2019

**DOI:** 10.1111/dom.70025

**Published:** 2025-09-01

**Authors:** Naomi Holman, Bob Young, Edward W. Gregg, Nick Wareham, Stephen Sharp, Kamlesh Khunti, Naveed Sattar, Jonathan Valabhji

**Affiliations:** ^1^ School of Public Health Imperial College London UK; ^2^ School of Population Health Royal College of Surgeons in Ireland Dublin Ireland; ^3^ Diabetes UK London UK; ^4^ MRC Epidemiology Unit, Institute of Metabolic Science University of Cambridge, Cambridge Biomedical Campus Cambridge UK; ^5^ Diabetes Research Centre University of Leicester Leicester UK; ^6^ School of Cardiovascular and Metabolic Health University of Glasgow Glasgow UK; ^7^ Division of Metabolism, Digestion and Reproduction, Faculty of Medicine Imperial College London London UK; ^8^ Beta Cell Centre, Chelsea & Westminster Hospital NHS Foundation Trust London UK; ^9^ Diabetes Programme, NHS England London UK

**Keywords:** heart failure, macrovascular disease, real‐world evidence, type 2 diabetes

## Abstract

**Aims:**

To assess longitudinal trends in total and cause‐specific mortality rates and in hospitalisation rates for diabetes complications among people with type 2 diabetes in England between 2009 and 2019; and to assess how trends differ by patient characteristics.

**Materials and Methods:**

A sequential cohort study of people with type 2 diabetes aged ≥20 years was performed using data from the National Diabetes Audit. Discretised Poisson regression models, adjusted for age, sex, ethnicity, socio‐economic deprivation and diabetes duration, were used to calculate total and cause‐specific mortality rates, as well as hospitalisation rates for myocardial infarction, stroke, heart failure, kidney and liver disease.

**Results:**

Total mortality declined in people aged 20–74 years (rate ratio [RR] 0.96, 95% CI 0.95–0.97) and aged ≥75 years (0.93, 0.92–0.94) between 2009–2011 and 2018–2019, predominantly due to reductions in cardiovascular deaths. Over the same time period, in those aged 20–74 years, total mortality declined in people of South Asian (0.92:0.0.87–0.96) but was unchanged in people of White (1.00: 0.99–1.01) ethnicities. Total mortality declined more in people living in the least (0.91:0.88–0.94) compared to the most (0.97:0.95–1.00) deprived areas. A composite endpoint of cardiovascular hospitalisations and mortality increased between 2009–2011 and 2018–2019 in those aged 20–49 years (1.20:1.14–1.27) and 50–74 years (1.04:1.03–1.05) but declined in those aged ≥75 years (0.85:0.84–0.86). Rates of hospitalisation for kidney and liver disease increased in all age groups.

**Conclusions:**

By examining longitudinal trends in mortality and hospitalisations according to different characteristics in people with type 2 diabetes in England, we have identified important targets for improvement through changes in health policy and care delivery.

## 1 INTRODUCTION

In 2012, the National Audit Office assessed the quality and costs of the management of adult diabetes services in the National Health Service (NHS) in England, and made recommendations for improvement^1^; a progress report followed in 2015.^2^ To assess the impact of resulting additional investment and work programmes, and to inform future plans, the NHS England Diabetes Programme commissioned this study to assess associated changes in national longitudinal trends in type 2 diabetes complications and mortality.

Over the past 30 years, mortality declines have occurred in the general population in England^3^ and in those with diabetes in other countries^4^, ^5^ strongly contributed to by declining cardiovascular deaths. However, during the 2010s, there was a slowing of these long‐term downward trends,^6^ plus evidence of increasing inequality in mortality by socio‐economic status.^7^

Recent studies, using data derived from a subset of the UK population, have suggested reductions in cardiovascular hospitalisations and mortality in people with diabetes between 2001 and 2018,^8^, ^9^ with evidence of diversification of the causes of mortality and morbidity.^10^ International evidence suggests that the declines in diabetes complications seen in the past 30 years have not been sustained^11^, ^12^ with divergence of trends by age with greater improvements in older people.^11^ However, previous studies have not examined trends by demographic characteristics.

Using the National Diabetes Audit (NDA) linked to hospitalisations and death registrations, we assessed changes in total and cause‐specific mortality and hospitalisations for myocardial infarction, stroke, heart failure, kidney disease, and liver disease from 2009 to 2019 in adults with type 2 diabetes in England.

## 2 MATERIALS AND METHODS

### 2.1 Study cohort and observation periods

Since 2003, the NDA has collated data annually on people registered with a general practice in England who have a valid code for diabetes mellitus (excluding gestational diabetes) in their electronic health record.^13^ Demographic and clinical data are extracted from the electronic health records by the General Practice Extraction Service and supplemented with data submitted by specialist diabetes services. Annual data collections cover a 15‐month period from 1 January in the first year to 31 March in the subsequent year. Hospital Episode Statistics (HES) record all NHS inpatient episodes; civil death registrations are recorded by the Office for National Statistics (ONS). These data sources are linked through a unique patient identifier, the NHS number.

Type 2 diabetes was identified from diagnostic codes.^13^ Where a specialist service had reported one or more diagnostic codes, the latest reported type of diabetes was used. Where no data had been provided by a specialist service, the latest type of diabetes recorded in the general practice electronic health record was used.

Sequential cohorts of people aged ≥20 years with type 2 diabetes were identified. The first annual cohort identified people in the 2006/07 NDA cohort (on whom data had been collected between 1 January 2006 and 31 March 2007) who were still alive on 1 January 2009. The observation period for this cohort was 1 January 2009 to 31 December 2009. Subsequent annual cohorts were identified, with the final cohort including people from the 2016/2017 NDA data collection (1 January 2016 to 31 March 2017) still alive on 1 January 2019, with an observation period from 1 January 2019 to 31 December 2019. People were included in multiple annual cohorts if they appeared in multiple NDA data collections and as they progressed through life; there was no linkage of individuals across the sequential cohorts in these analyses. Since 2016, NDA general practice participation has exceeded 95%; although in earlier years participation was lower, related to organisational factors geographically within the health service, rather than due to patient characteristics, so that data have historically been considered representative. Tables S1 and S2 demonstrate the number of people included and the percentage general practice participation in each audit year.

For this study, annual cohorts were combined to create four observation time periods—2009–2011, 2012–2014, 2015–2017, and 2018–2019. The time period of 2009–2011 was treated as the reference period for all outcomes except kidney disease; due to changes in coding, the reference period for kidney disease was from 2012 to 2014.

### 2.2 Outcomes

Outcomes were total and cause‐specific mortality, and NHS hospitalisations for myocardial infarction, stroke, heart failure, kidney disease and liver disease. Hospitalisation was defined as an inpatient stay of at least one night; diagnostic codes are allocated at the end of the episode of care. Primary underlying cause of death was split into cardiovascular disease (ICD‐10 codes I00‐99), cancer (C00‐97), kidney disease (N00‐28), liver disease (K70‐77), respiratory disease (J01‐99), infections (A00‐99, B00‐99), dementia (F00‐03, G30), diabetes (E10‐14) and, by exclusion, other causes. Categories were based on the leading causes of death used by ONS, aggregated appropriately for small numbers and to reflect relevance to type 2 diabetes. Hospitalisations were defined by primary diagnosis for the admission (myocardial infarction [I21‐22], stroke [I61, I63‐4, I679], heart failure [I50], kidney disease [N00‐28] and liver disease [K70‐77]). A composite measure of cardiovascular disease, consisting of deaths from cardiovascular disease plus hospitalisations for myocardial infarction, stroke and heart failure, was also analysed. The sequential cohort design with a 1‐year follow‐up period means that individuals are only counted once if they have multiple hospital admissions that year, whilst hospital admissions in subsequent years are counted within those time periods.

### 2.3 Exposures

Age and duration of diabetes were calculated on 1st January for each year. Where a valid ethnicity was recorded in the NDA, this was used; otherwise, the latest valid ethnicity recorded in HES was used. Ethnicity was grouped into South Asian, Black, Mixed, Other, or White. Socio‐economic deprivation was identified by linking individuals' home postcodes to the Indices of Multiple Deprivation^14^, ^15^, ^16^ grouped into quintiles for analysis.

### 2.4 Statistical analysis

Poisson regression models with mortality or hospitalisation as the outcome variable and age, sex, deprivation, ethnicity and diabetes duration as explanatory variables, with time in years as the off‐set variable, were created. As the outcomes of interest were count data, Poisson regression models were chosen as they allow an assessment of event rates over time and there is an assumption of independence within each model. The assumptions underlying Poisson regression models were assessed and overdispersion measured using Pearson's chi‐squared tests: the results were acceptable. These models provided the adjusted rate ratio (RR) for the time periods 2012–2014, 2015–2017 and 2018–2019 compared to 2009–2011.

The analyses of mortality were stratified into two age groups (20–74 years, and ≥75 years, in line with the UK government definition of premature mortality) and the analyses of hospitalisations were stratified into three age groups (20–49 years, 50–74 years and ≥75 years); the event rate for mortality was too low in the 20–49 age range to be analysed separately. To assess whether changes in mortality or hospitalisation rates varied by sex, ethnicity, socio‐economic deprivation, and diabetes duration, models with an interaction term of sex by time period, ethnicity by time period, quintile of deprivation by time period, and diabetes duration by time period were created. These analyses were stratified into the two age categories (20–74 years and ≥75 years). No censoring occurred in the analyses of mortality, but for hospitalisations, individuals were censored if they died during the follow‐up period (Table S3). All categorical variables included a category of “missing”, so no imputation was undertaken.

The intercepts and coefficients from the Poisson regression models were used to calculate mortality and hospitalisation rates for the time periods 2009–2011, 2012–2014, 2015–2017 and 2018–2019 for a population matching the characteristics of the 2 535 630 people with type 2 diabetes who were the latest cohort included in the 2016/17 NDA and still alive on 1st January 2019.

The legal bases for NDA data collection and analyses, and associated information governance, have been outlined recently,^17^ and are detailed in Supporting Information page 13.

## 3 RESULTS

### 3.1 Characteristics of cohorts

A total of 3 530 070 people were followed for 17 762 145 person‐years. The median age of the cohort changed from 68.0 years (IQR 58.5–76.5) in 2009–2011 to 68.4 (IQR 58.3–77.3) in 2018–2019. The proportion of people diagnosed with type 2 diabetes for <5 years decreased from 704 965 (41.9%) in 2009–2011 to 756 400 (28.0%) in 2018–2019. The proportion diagnosed for ≥15 years increased from 182 290 (10.8%) to 543 260 (20.1%) (Table 1).

**TABLE 1:** Characteristics of people included in the 2009–2011, 2012–2014, 2015–2017, and 2018–2019 cohorts.

	2009–2011		2012–2014		2015–2017		2018–2019	
	*n*	%	*n*	%	*n*	%	*n*	%
Persons	1 680 660		2 263 440		2 236 920		2 697 755	
Women	757 725	45.1	1 005 630	44.4	989 715	44.2	1 190 130	44.1
Men	922 935		1 257 810		1 247 205		1 507 625	
Age at start of time period								
20–29 years	6575	0.4	9400	0.4	8720	0.4	10 195	0.4
30–39 years	40 750	2.4	55 015	2.4	51 705	2.3	62 060	2.3
40–49 years	152 030	9.0	207 740	9.2	195 245	8.7	226 070	8.4
50–59 years	307 945	18.3	422 990	18.7	420 340	18.8	511 615	19.0
60–69 years	454 650	27.1	612 040	27.0	596 670	26.7	693 280	25.7
70–79 years	462 050	27.5	592 820	26.2	582 165	26.0	709 960	26.3
≥80 years	256 660	15.3	363 440	16.1	382 075	17.1	484 580	18.0
Median (IQR)	68.0 (58.5–76.5)		68.0 (58.4–76.9)		68.2 (58.3–77.2)		68 (58.3–77.3)	
Deprivation								
Most deprived	432 250	25.7	555 445	24.5	551 875	24.7	703 635	26.1
2nd most deprived	363 175	21.6	486 795	21.5	484 935	21.7	620 525	23.0
3rd most deprived	342 905	20.4	454 330	20.1	444 045	19.9	559 885	20.8
2nd least deprived	299 780	17.8	410 265	18.1	400 570	17.9	477 975	17.7
Least deprived	235 245	14.0	355 745	15.7	346 305	15.5	330 055	12.2
Missing	7305	0.4	860	0.0	9190	0.4	5680	0.2
Ethnicity								
White	1 346 237	80.1	1 789 608	79.1	1 743 923	78.0	2 045 124	75.8
Mixed	13 803	0.8	20 132	0.9	21 325	1.0	28 787	1.1
South Asian	156 172	9.3	215 337	9.5	222 685	10.0	299 076	11.1
Black	66 395	4.0	97 449	4.3	103 306	4.6	131 293	4.9
Other	66 338	3.9	96 986	4.3	98 760	4.4	129 011	4.8
Missing	31 714	1.9	43 929	1.9	46 922	2.1	64 464	2.4
Duration of diagnosis								
< 5 years	704 965	41.9	866 810	38.3	714 415	31.9	756 400	28.0
5–9·9 years	558 185	33.2	730 345	32.3	682 310	30.5	784 775	29.1
10–14·9 years	224 955	13.4	384 745	17.0	501 715	22.4	611 495	22.7
≥15 years	182 290	10.8	273 100	12.1	334 785	15.0	543 260	20.1
Missing	10 265	0.6	8440	0.4	3695	0.2	1820	0.1
Median (IQR)	7.0 (4.4–11.0)	7.9 (4.5–12.0)			8.7 (5.0–13.4)		9.4 (5.3–14.5)	

### 3.2 All‐cause mortality

**FIGURE 1:**
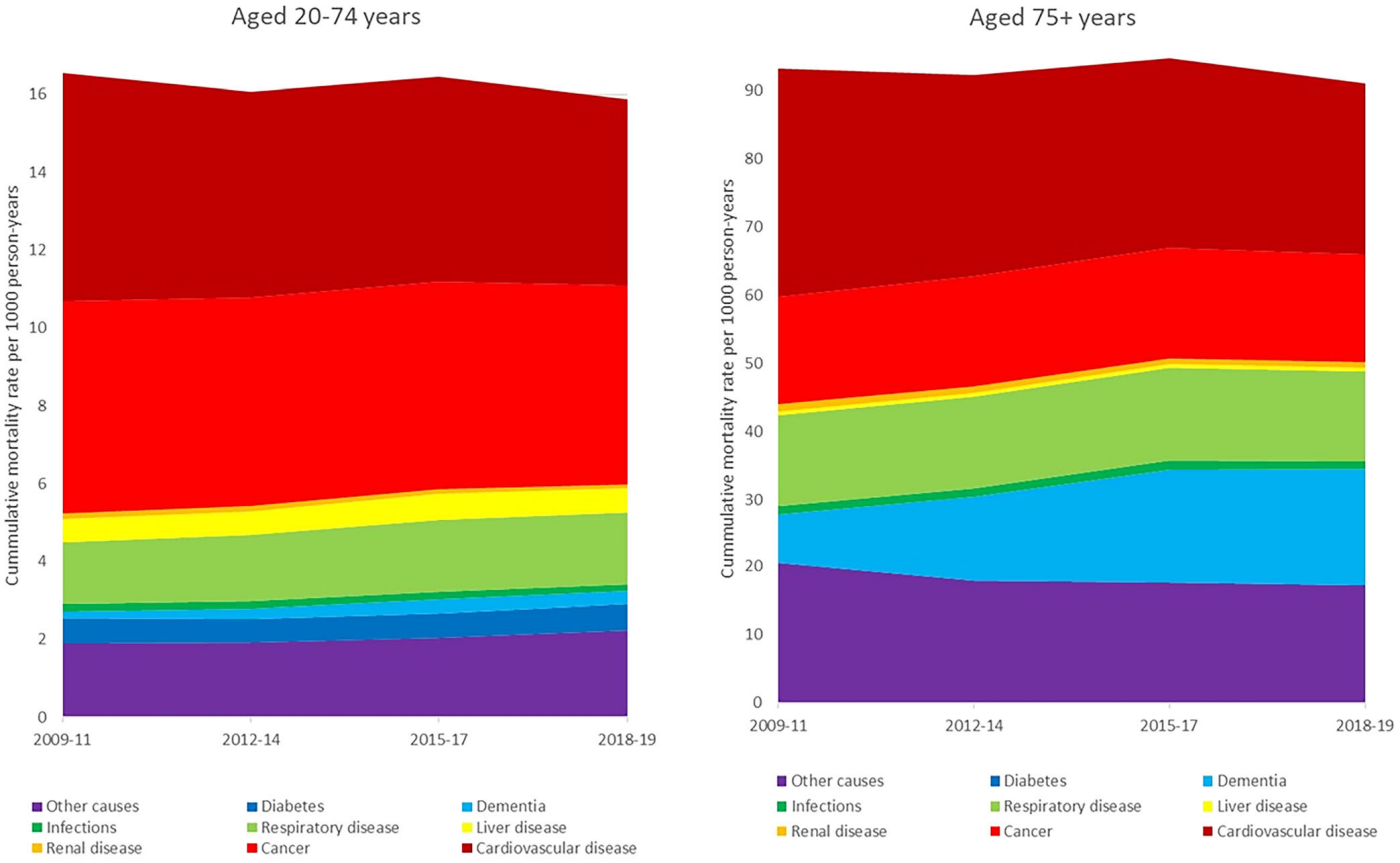
Cumulative mortality rates by cause adjusted for age, sex, ethnicity, deprivation and duration of diagnosed diabetes for people aged 20–74 years and aged 75 years and older.

Adjusted total mortality declined between 2009–2011 and 2018–2019 in people aged 20–74 years (16.6 to 15.9 per 1000 person‐years, RR 0.96, 95% CI 0.95–0.97) (Figure 1, Figure S1, Tables S4A and S5) and in people aged ≥75 years (93.4–91.1 per 1000 person‐years, RR 0.98 95% CI 0.97–0.98) (Figure 1, Figure S1, Tables S4B and S5). In both age groups, total mortality was lower in women than in men, lower in those of South Asian, Black, Mixed and Other ethnicities compared to those of White ethnicity, and lower in those with a shorter diabetes duration. In both age groups, there was a socio‐economic deprivation gradient with lower total mortality in each quintile compared to the adjacent quintile, as deprivation decreased. The relative differences in total mortality by ethnicity and deprivation were greater in the 20–74 age group compared to the ≥75 age group (Table S4A,B).

### 3.3 Cause specific mortality

Between 2009–2011 and 2018–2019, there were decreases in adjusted mortality for cardiovascular disease and kidney disease in those aged 20–74 years and aged ≥75 years (Figure 1, Table S4A,B). In the younger cohort, there were reductions in cancer and infections and increases in respiratory disease and dementia, but no change in liver disease or diabetes specific mortality. In the older cohort, diabetes specific mortality declined and liver disease and dementia increased, but cancer, respiratory disease and infections were unchanged.

In both age groups, adjusted mortality for cardiovascular disease, cancer and liver disease was lower in women than men, and for dementia, higher in women than men. In the younger group, mortality from diabetes‐specific causes and in the older group, kidney disease and respiratory disease were lower in women than men. Lower mortality for those of South Asian, Black, Mixed and Other ethnicities and for those living in the least deprived areas was found in all eight specific causes of mortality analysed, except for infections in the Mixed, South Asian and Other ethnic groups, and for diabetes‐specific causes in the Mixed ethnic group (Table S4A,B). The top 10 causes of death based on counts of individual deaths for each age group in each time period are shown in Table S10, which shows that myocardial infarction is the most common individual cause of death in those aged 20–74 years in all time periods. In those aged 75 years and older, chronic ischaemic heart disease is the most common cause of death in 2009–2011 and 2013–2015, but unspecified dementia in the latter two time periods.

### 3.4 Variation in trends in mortality

There were statistically significant interactions between total mortality and changes over time by ethnicity, deprivation, diabetes duration and sex (Tables S6–S9). Examination of the stratified relative risks for each time period for people aged 20–74 years showed no change in total mortality in those of White ethnicity (RR 1.00 95% CI 0·99–1.01) but a decline in those of South Asian ethnicity (RR 0.92:0.87–0.96) (Table S6). In those aged 20–74 years, people living in the least deprived quintile experienced a greater decline in total mortality between 2009–2011 and 2018–2019 (RR 0.91, 95% CI 0.88–0.94) than those living in the most deprived quintile (RR 0.97, 95% CI 0.95–1.00) (Table S7). In the same age group, having diabetes duration <10 years was associated with declining total mortality, and duration ≥10 years increasing total mortality (Table S8). In both age groups, there were statistically significant interactions between changes in total mortality over time by sex, but overlapping confidence intervals between women and men meant no clear differing trends could be identified (Table S9).

### 3.5 Hospitalisations for myocardial infarction, stroke, heart failure, kidney disease and liver disease

**FIGURE 2:**
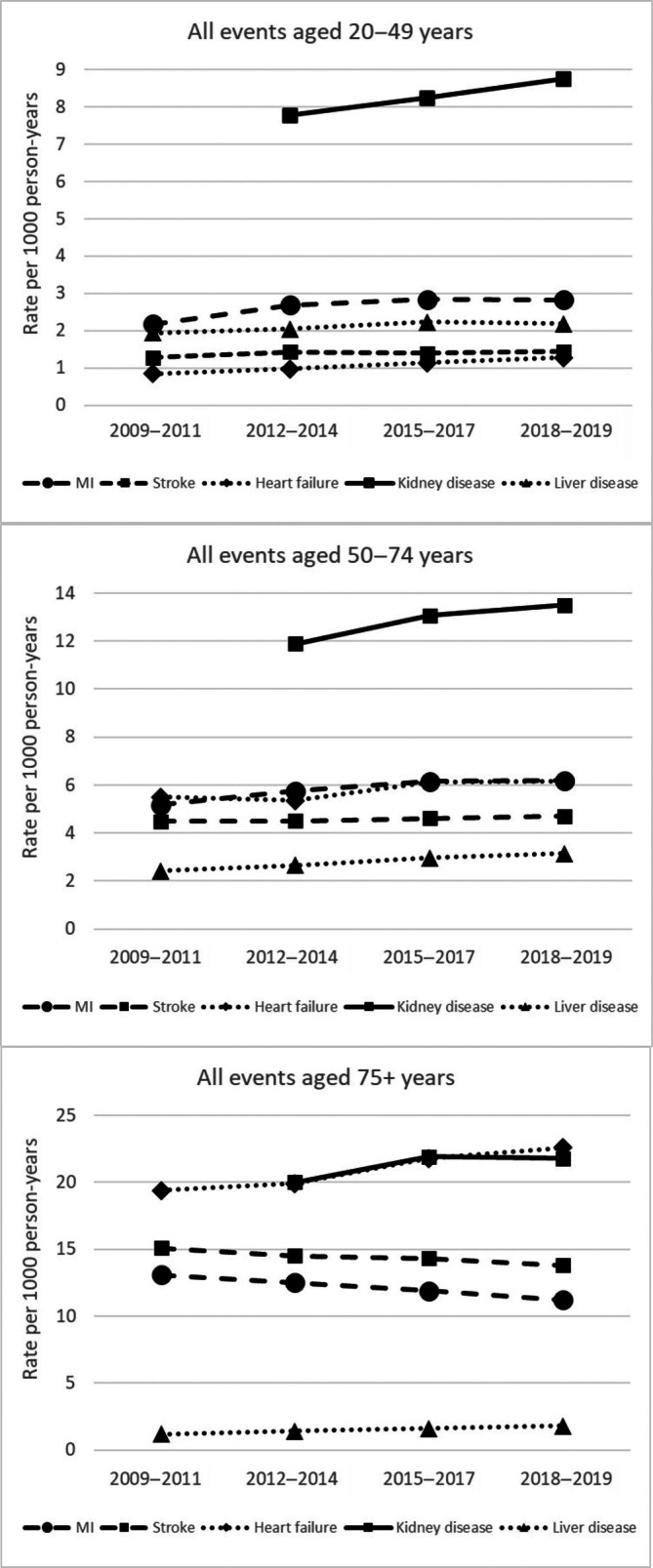
Trends in hospitalisation rates for myocardial infarction, stroke, heart failure, kidney disease and liver disease adjusted for age, sex, ethnicity, socio‐economic deprivation and diabetes duration for people aged 20–49 years, 50–74 years and 75 years and older.

Among those aged 20–49 years and 50–74 years, rates of hospitalisations for myocardial infarction, stroke, heart failure, and liver disease increased significantly between 2009–2011 and 2018–2019, whilst the rate of hospitalisation for kidney disease increased between 2012–2014 and 2018–2019 (Figure 2, Table 2). Among people aged ≥75 years, rates of hospitalisations for myocardial infarction and stroke decreased between 2009–2011 and 2018–2019, whilst heart failure and liver disease increased, and between 2012–2014 and 2018–2019, kidney disease increased (Figure 2, Table 2).

**TABLE 2:** Rate ratios for hospital admissions for myocardial infarction, stroke, heart failure, cardiovascular composite indicator, kidney disease and liver disease.

	MI	Stroke	Heart failure	Composite indicator of cardiovascular disease	Kidney disease	Liver disease
	RR (95% CI)	RR (95% CI)	RR (95% CI)	RR (95% CI)	RR (95% CI)	RR (95% CI)
**Age 20–49 years**						
Age (years)						
Per additional year	1.08 (1.08–1.09)	1.07 (1.06–1.08)	1.08 (1.07–1.09)	1.08 (1.07–1.08)	1.02 (1.01–1.02)	1.02 (1.02–1.03)
Sex						
Men	1.00	1.00	1.00	1.00	1.00	1.00
Women	0.55 (0.51–0.58)	0.97 (0.9–1.05)	0.72 (0.66–0.79)	0.68 (0.65–0.71)	1.08 (1.04–1.12)	0.72 (0.67–0.76)
Deprivation						
Most deprived	1.00	1.00	1.00	1.00	1.00	1.00
2nd most deprived	0.81 (0.76–0.87)	0.93 (0.84–1.02)	0.82 (0.74–0.92)	0.85 (0.81–0.89)	0.92 (0.88–0.97)	0.85 (0.78–0.92)
3rd most deprived	0.70 (0.64–0.76)	0.76 (0.68–0.85)	0.70 (0.61–0.79)	0.72 (0.68–0.76)	0.91 (0.86–0.95)	0.77 (0.70–0.84)
2nd least deprived	0.60 (0.55–0.67)	0.68 (0.59–0.77)	0.64 (0.55–0.74)	0.63 (0.59–0.67)	0.81 (0.77–0.86)	0.71 (0.64–0.78)
Least deprived	0.55 (0.49–0.61)	0.62 (0.54–0.72)	0.49 (0.41–0.59)	0.55 (0.51–0.59)	0.76 (0.71–0.81)	0.69 (0.62–0.78)
Missing	0.67 (0.47–0.96)	0.57 (0.33–0.99)	0.79 (0.48–1.31)	0.72 (0.57–0.90)	1.02 (0.85–1.24)	0.83 (0.57–1.19)
Ethnicity						
White	1.00	1.00	1.00	1.00	1.00	1.00
Mixed	0.67 (0.52–0.85)	0.77 (0.56–1.05)	0.81 (0.58–1.13)	0.72 (0.61–0.83)	0.99 (0.87–1.13)	0.53 (0.40–0.71)
South Asian	1.05 (0.98–1.12)	0.70 (0.63–0.78)	0.53 (0.47–0.60)	0.75 (0.72–0.79)	0.98 (0.94–1.02)	0.51 (0.47–0.56)
Black	0.34 (0.29–0.40)	0.92 (0.80–1.06)	0.99 (0.85–1.15)	0.62 (0.57–0.67)	0.91 (0.85–0.98)	0.39 (0.33–0.45)
Other	0.72 (0.64–0.81)	0.72 (0.61–0.84)	0.58 (0.48–0.70)	0.63 (0.58–0.69)	0.94 (0.88–1.01)	0.55 (0.48–0.63)
Missing	0.09 (0.05–0.16)	0.11 (0.05–0.22)	0.02 (0.00–0.13)	0.29 (0.24–0.36)	0.06 (0.04–0.10)	0.08 (0.04–0.15)
Duration of diabetes						
Per additional year	1.01 (1.009–1.012)	1.01 (1.008–1.012)	1.012 (1.01–1.014)	1.011 (1.01–1.012)	1.01 (1.009–1.011)	1.005 (1.002–1.007)
Time period						
2009–11	1.00	1.00	1.00	1.00	‐	1.00
2012–14	1.24 (1.14–1.35)	1.11 (1.00–1.24)	1.14 (1.00–1.31)	1.13 (1.07–1.20)	1.00	1.05 (0.96–1.15)
2015–17	1.31 (1.20–1.43)	1.08 (0.96–1.22)	1.34 (1.17–1.54)	1.19 (1.12–1.26)	1.06 (1.01–1.11)	1.15 (1.05–1.26)
2018–19	1.30 (1.19–1.42)	1.12 (1.00–1.26)	1.50 (1.32–1.72)	1.20 (1.14–1.27)	1.13 (1.08–1.17)	1.12 (1.02–1.23)
**Age 50–74 years**						
Age						
Per additional year	1.03 (1.03–1.03)	1.06 (1.06–1.06)	1.08 (1.08–1.08)	1.06 (1.06–1.06)	1.02 (1.02–1.02)	0.99 (0.99–0.99)
Sex						
Men	1.00	1.00	1.00	1.00	1.00	1.00
Women	0.57 (0.56–0.58)	0.76 (0.75–0.78)	0.75 (0.74–0.77)	0.67 (0.66–0.68)	0.83 (0.82–0.84)	0.86 (0.84–0.88)
Deprivation						
Most deprived	1.00	1.00	1.00	1.00	1.00	1.00
2nd most deprived	0.87 (0.85–0.89)	0.86 (0.84–0.88)	0.81 (0.79–0.82)	0.84 (0.83–0.85)	0.88 (0.87–0.90)	0.91 (0.88–0.94)
3rd most deprived	0.76 (0.75–0.78)	0.76 (0.74–0.78)	0.68 (0.67–0.70)	0.72 (0.71–0.73)	0.80 (0.78–0.81)	0.86 (0.83–0.89)
2nd least deprived	0.68 (0.66–0.69)	0.68 (0.66–0.70)	0.57 (0.55–0.58)	0.63 (0.62–0.64)	0.74 (0.72–0.75)	0.83 (0.80–0.86)
Least deprived	0.60 (0.58–0.62)	0.61 (0.59–0.63)	0.49 (0.48–0.50)	0.56 (0.55–0.56)	0.71 (0.69–0.72)	0.75 (0.73–0.78)
Missing	0.78 (0.70–0.87)	0.77 (0.68–0.87)	0.89 (0.80–0.98)	0.82 (0.77–0.87)	0.92 (0.86–1.00)	1.20 (1.05–1.37)
Ethnicity						
White	1.00	1.00	1.00	1.00	1.00	1.00
Mixed	0.83 (0.76–0.91)	0.95 (0.87–1.05)	0.86 (0.79–0.94)	0.83 (0.79–0.88)	1.01 (0.95–1.07)	0.56 (0.48–0.64)
South Asian	1.45 (1.41–1.48)	0.95 (0.92–0.98)	1.06 (1.04–1.09)	1.03 (1.02–1.05)	1.08 (1.06–1.1)	0.56 (0.54–0.59)
Black	0.52 (0.50–0.55)	1.01 (0.97–1.05)	0.84 (0.81–0.88)	0.73 (0.72–0.75)	1.05 (1.02–1.08)	0.36 (0.33–0.39)
Other	0.90 (0.86–0.93)	0.84 (0.80–0.88)	0.82 (0.79–0.86)	0.81 (0.79–0.83)	0.95 (0.92–0.97)	0.71 (0.67–0.75)
Missing	0.11 (0.10–0.13)	0.15 (0.13–0.17)	0.09 (0.07–0.10)	0.40 (0.38–0.42)	0.09 (0.08–0.1)	0.11 (0.09–0.14)
Duration of diabetes						
Per additional year	1.03 (1.03–1.03)	1.06 (1.06–1.06)	1.08 (1.08–1.08)	1.06 (1.06–1.06)	1.02 (1.02–1.02)	0.99 (0.99–0.99)
Time period						
2009–11	1.00	1.00	1.00	1.00	‐	1.00
2012–14	1.11 (1.09–1.14)	1.01 (0.98–1.03)	0.97 (0.95–0.99)	0.99 (0.98–1.01)	1.00	1.09 (1.05–1.13)
2015–17	1.19 (1.16–1.22)	1.03 (1.00–1.06)	1.11 (1.09–1.14)	1.05 (1.04–1.07)	1.10 (1.08–1.12)	1.22 (1.18–1.27)
2018–19	1.20 (1.17–1.23)	1.05 (1.02–1.08)	1.12 (1.09–1.14)	1.04 (1.03–1.05)	1.14 (1.12–1.15)	1.30 (1.25–1.34)
**Age 75 years and older**						
Age						
Per additional year	1.05 (1.04–1.05)	1.06 (1.06–1.06)	1.06 (1.06–1.06)	1.07 (1.07–1.07)	1.03 (1.03–1.03)	0.93 (0.92–0.93)
Sex						
Men	1.00	1.00	1.00	1.00	1.00	1.00
Women	0.71 (0.70–0.72)	0.98 (0.96–0.99)	0.81 (0.80–0.82)	0.81 (0.80–0.81)	0.70 (0.70–0.71)	0.80 (0.76–0.83)
Deprivation						
Most deprived	1.00	1.00	1.00	1.00	1.00	1.00
2nd most deprived	0.95 (0.92–0.97)	0.96 (0.94–0.98)	0.92 (0.90–0.94)	0.94 (0.93–0.95)	0.92 (0.90–0.93)	0.89 (0.83–0.95)
3rd most deprived	0.89 (0.87–0.91)	0.93 (0.91–0.96)	0.82 (0.81–0.84)	0.88 (0.87–0.89)	0.83 (0.81–0.85)	0.82 (0.77–0.88)
2nd least deprived	0.86 (0.84–0.88)	0.91 (0.89–0.93)	0.77 (0.75–0.78)	0.85 (0.84–0.85)	0.78 (0.76–0.79)	0.87 (0.81–0.93)
Least deprived	0.79 (0.77–0.81)	0.88 (0.86–0.91)	0.70 (0.68–0.71)	0.78 (0.78–0.79)	0.72 (0.71–0.74)	0.79 (0.73–0.85)
Missing	0.88 (0.79–0.99)	0.94 (0.85–1.04)	0.80 (0.73–0.87)	0.85 (0.81–0.90)	1.00 (0.91–1.09)	1.12 (0.86–1.47)
Ethnicity						
White	1.00	1.00	1.00	1.00	1.00	1.00
Mixed	0.77 (0.69–0.86)	0.95 (0.87–1.05)	0.98 (0.91–1.06)	0.88 (0.84–0.93)	1.10 (1.01–1.18)	0.67 (0.49–0.92)
South Asian	1.52 (1.47–1.56)	0.94 (0.91–0.97)	1.28 (1.25–1.31)	1.10 (1.09–1.12)	1.05 (1.02–1.08)	0.91 (0.83–1.00)
Black	0.60 (0.57–0.63)	0.95 (0.91–0.99)	0.92 (0.89–0.95)	0.81 (0.79–0.83)	1.11 (1.07–1.15)	0.60 (0.52–0.69)
Other	1.08 (1.03–1.13)	0.92 (0.88–0.96)	1.03 (0.99–1.07)	0.98 (0.96–1.01)	1.02 (0.98–1.06)	0.96 (0.84–1.09)
Missing	0.22 (0.19–0.25)	0.30 (0.27–0.34)	0.18 (0.16–0.21)	0.58 (0.56–0.60)	0.16 (0.14–0.18)	0.35 (0.25–0.48)
Duration of diabetes						
Per additional year	1.005 (1.005–1.006)	1.004 (1.004–1.004)	1.006 (1.005–1.006)	1.005 (1.005–1.005)	1.008 (1.007–1.008)	1.005 (1.003–1.006)
Time period						
2009–11	1.00	1.00	1.00	1.00	‐	1.00
2012–14	0.95 (0.93–0.97)	0.96 (0.94–0.98)	1.03 (1.01–1.04)	0.94 (0.93–0.95)	1.00	1.19 (1.11–1.27)
2015–17	0.91 (0.89–0.93)	0.95 (0.93–0.97)	1.12 (1.10–1.14)	0.94 (0.93–0.95)	1.09 (1.08–1.11)	1.40 (1.31–1.5)
2018–19	0.85 (0.83–0.87)	0.91 (0.89–0.93)	1.16 (1.14–1.18)	0.90 (0.89–0.91)	1.09 (1.07–1.11)	1.49 (1.39–1.6)

*Note*: Additional decimal places are shown for duration of diabetes due to the small unit increases and the narrow ranges.

In the 20–49 age group, people of South Asian ethnicity had lower rates of hospitalisations for stroke, heart failure and liver disease, whilst those of Black ethnicity had lower rates for myocardial infarction, kidney disease and liver disease compared to those of White ethnicity (Table 2). People aged 50–74 years of South Asian ethnicity had higher rates of hospitalisations for myocardial infarction and heart failure but lower rates for stroke and liver disease than those of White ethnicity, while those of Black ethnicity had lower rates of hospitalisations for myocardial infarction, heart failure and liver disease (Table 2). People aged ≥75 years of South Asian ethnicity had higher rates of hospitalisations for myocardial infarction, heart failure and kidney disease and lower rates for stroke and liver disease, while those of Black ethnicity had lower rates for myocardial infarction, stroke and heart failure and higher rates for kidney disease (Table 2).

### 3.6 Variation in trends in hospitalisations

The models including the interaction terms (Tables S6–S9) for people aged 20–74 years showed that longer diabetes duration was associated with greater increases in hospitalisations for myocardial infarction, stroke, heart failure, and kidney disease over time. The greatest difference in time trends was found in hospitalisation for heart failure, where the RR for 2018–2019 compared to 2009–2011 was 1.14 (95% CI 1.08–1.21) for those with a diagnosis of <5 years compared to 1.57 (95% CI 1.49–1.65) for those diagnosed 10–14.9 years and 2.34 (95% CI 2.22–2.47) for those diagnosed ≥15 years. Similar changes over time were seen for hospitalisations for myocardial infarction, stroke, and kidney disease. There was no change in hospitalisation for liver disease over time for those diagnosed <5 years (RR 0.47, 95% CI 0.11–1.94) but statistically significant increases for those with longer diabetes duration (RR 1.12 95% CI 1.06–1.19 for those diagnosed 5–9.9 years, RR 1.31 95% CI 1.23–1.39 for those diagnosed 10–14.9 years and 1.42 95% CI 1.3301.52 for those diagnosed ≥15 years). Greater relative increases in hospitalisations for myocardial infarction and liver disease were found in those living in the least deprived areas compared to those in the most deprived (RR 1.30, 95% CI 1.21–1.39 compared to 1.14, 95% CI 1.10–1.19 and RR 1.37, 95% CI 1.25–1.50 compared to 1.17, 95% CI 1.10–1.24 for myocardial infarction and liver disease, respectively) (Table S7). The differences in changes over time in hospitalisations for stroke, heart failure and kidney disease were not statistically significant. There were no statistically significantly different trends over time by sex or ethnicity.

### 3.7 Composite indicator of cardiovascular disease

Between 2009–2011 and 2018–2019, the risk of cardiovascular disease, as defined by the composite indicator, increased in those aged 20–49 years (RR 1.20, 95% CI 1.14–1.27) and aged 50–74 years (RR 1.04, 95% CI 1.03–1.05) but declined in those aged ≥75 years (RR 0.90, 95% CI 0.89–0.91) (Table 2).

## 4 CONCLUSIONS

In this national study of over 3.5 million adults with type 2 diabetes, we found that, after adjustment for the changing demographic characteristics and diabetes duration, between 2009 and 2019, relative reductions in total mortality rates were similar in those aged 20–74 years and in those aged ≥75 years; although the absolute rate reduction was greater in the older group. There were greater relative reductions in mortality in those of South Asian ethnicity compared to those of White ethnicity and in those living in less deprived areas compared to more deprived areas, findings mirroring those in the general population.^7^, ^18^, ^19^, ^20^, ^21^

Mortality is low at younger ages, so that patterns of morbidity are more sensitive to changes over time in younger cohorts. Cardiovascular hospitalisations increased in those aged <75 years, increasing most in the youngest cohort aged 20–49 years, and decreased in those aged ≥75 years. This is consistent with other emerging evidence.^22^ Analyses of NDA data consistently show that younger people with diabetes are less likely to receive annual care processes or achieve treatment targets for HbA1c, blood pressure and cholesterol,^23^ and this may now be impacting clinical endpoints. In addition, hospitalisations for kidney and liver disease increased across all ages. An international review of diabetes complications up to 2015 found that in high‐income countries the rate of macrovascular disease had declined but that end‐stage kidney disease had increased.^11^ A recent analysis of people with diabetes in the United States suggested a resurgence in the incidence of hospital admissions for myocardial infarction and stroke in those aged <65 years, but not in those aged ≥65 years, starting around 2009. Concurrently, the incidence of hospitalisation for end‐stage kidney disease rose in those aged 18–44 years.^10^ An Australian study over a similar time also noted that any improvements in cardiovascular incidence were largely limited to those aged ≥60 years. Similarly, reductions in mortality rates were observed in people with type 2 diabetes in Australia in men aged ≥40 years and women aged ≥60 years but not at younger ages between 2002 and 2014.^24^, ^25^ Collectively, such data highlight the need for greater preventative efforts in earlier‐onset type 2 diabetes.

Mortality rates in those of South Asian, Black, Mixed and other ethnicities were lower than those of White ethnicity across almost all causes of death including cardiovascular disease, despite a higher prevalence of cardiovascular disease among these groups compared to those of White ethnicity in the general population.^18^ Previous work has demonstrated more years of life lost among those of White ethnicity with diabetes than among those of South Asian or Black ethnicity,^19^ findings that now match mortality patterns in the general population.^20^ The reasons for these differences by ethnicity are likely to be multi‐factorial but may relate to a healthy migrant effect,^21^ or the need for relatively greater weight gain in those of White ethnicity in order to develop type 2 diabetes.^23^

The scale of the relative reduction in total mortality shown in this analysis (4% in those age 20–74 years and 2% in those aged ≥75 years over the decade) is smaller than found in those with diabetes in the UK between 2001 and 2018, where a 31%–32% reduction was seen over the 17‐year period.^9^ Similar analyses in Sweden between 1998 and 2014^25^ and Denmark between 1996 and 2016^26^ reported reductions of 21% and 25% per decade, respectively. The smaller reduction in our study may reflect the slowing in the decline in total mortality in the general population of England over the latter time period.^6^, ^27^

Increasing rates of hospitalisation for liver disease were seen in people with type 2 diabetes across all ages, a novel finding. This may relate to a higher prevalence of, or longer exposure to, excess adiposity particularly in those with younger onset.^28^ The continued reduction in mortality leading to greater lifetime exposure to hyperglycaemia and other risk factors may explain rising kidney‐related hospitalisations, seen across all age cohorts in this study. Notably, the relative increase in hospitalisations for myocardial infarction and liver disease in those aged 20–74 years increased more in those living in the least compared to the most deprived areas; this may be due to greater survival among those in the least deprived areas or that the baseline rate was lower to begin with.

The strength of these analyses lies in the large cohort size, with data accrued over a decade. However, there are a number of limitations. While general practice participation is almost complete latterly, this was not the case in earlier years, so that representativeness over the entire period cannot be guaranteed. The change to longer diabetes duration across the observation periods is greater than might be expected and may relate to greater accuracy of the date of diagnosis latterly. Follow‐up was limited to 2019 due to the onset of the COVID‐19 pandemic and its associated healthcare disruptions. These analyses have used hospitalisations for the five specified conditions; whilst this likely provides a reliable assessment of the occurrences of myocardial infarction and stroke, it likely captures only a proportion of exacerbations of heart failure, kidney disease, and liver disease, as many will not result in hospitalisation. The increases in hospitalisation rates for liver disease may partially reflect improvements in diagnosis in addition to changes in actual prevalence. Due to delays between the end of the audit period and data extraction, this analysis only includes people who survived 21 months after the end of each annual audit period; the sequential cohort design of the study minimises the impact of this, but mortality and hospitalisations in people shortly after diagnosis are not fully accounted for. These analyses relied on nationally collated data from electronic health records to identify causes of hospitalisations and death; whilst the use of this type of data facilitates large‐scale analyses, there is no scope to independently assess the validity of codes used and how the quality of this information may have varied over time. Changes to diagnostic practices and initiatives to identify undiagnosed type 2 diabetes may have contributed to changes in type 2 diabetes phenotype, and it is not possible to quantify the scale and impact of these effects. The reliability of diagnostic codes to identify the type of diabetes in electronic health records is never perfect, but our approach to ascertainment is pragmatic and consistent over time in order to maximise accuracy. Over the time period analysed, prescribing patterns will have changed with the advent of new therapies; however, the NDA did not include data on prescriptions until the 2017/2018 data collection, so this could not be incorporated into these analyses. The NDA does not collect information on lifestyle factors such as diet and exercise, so similarly, these risk factors could not be accounted for. The cohort size means that small differences between groups or small changes over time might be statistically significant and, while relevant at the population level, may be less relevant clinically at the individual patient level. We have run multiple models, which will increase the likelihood of statistically significant differences arising by chance.

By examining longitudinal trends in mortality and hospitalisations according to different characteristics in people with type 2 diabetes in England, we have identified important targets for improvement through changes in health policy and care delivery. The results of these analyses, following discussions with the NHS England Diabetes Programme team who commissioned the study, have contributed to the allocation of £14.5 million additional investment nationally to support better care for those with early‐onset type 2 diabetes.^29^

## AUTHOR CONTRIBUTIONS

All authors designed the study, interpreted the results, and contributed to the writing and editing of the manuscript. NH undertook the analysis and had access to the raw data. NH and JV took the final responsibility for the submission of the paper. NH is the guarantor of this work and, as such, had full access to all the data in the study and takes responsibility for the integrity of the data and the accuracy of the data analysis.

## CONFLICT OF INTEREST STATEMENT

KK has acted as a consultant, speaker, or received grants for investigator‐initiated studies for AstraZeneca, Bayer, Novartis, Novo Nordisk, Sanofi‐Aventis, Lilly, and Merck Sharp & Dohme, Boehringer Ingelheim, Oramed Pharmaceuticals, Roche, and Applied Therapeutics. NS has received grants from AstraZeneca, Boehringer Ingelheim, Novartis, and Roche Diagnostics, and consulting fees from Abbott Laboratories, Amgen, AstraZeneca, Boehringer Ingelheim, Eli Lilly, Hanmi Pharmaceuticals, Janssen, Merck Sharp & Dohme, Novartis, Novo Nordisk, Pfizer, Roche Diagnostics, and Sanofi. NH has received payments from AstraZeneca for participation in trials as a patient. KK (Chair), NH, BY, and JV are members of the NDA Research Group. JV was National Clinical Director for diabetes and obesity at NHS England from 2013 to September 2023.

## PEER REVIEW

The peer review history for this article is available at https://www.webofscience.com/api/gateway/wos/peer-review/10.1111/dom.70025.

## Supporting information


**Data S1.** Supporting information.

## Data Availability

The National Diabetes Audit data can be requested via the NHS England Data Access Request Service.
