# Supplementary material for: Trends in mortality and hospitalisations for cardiovascular, kidney and liver disease in people with type 2 diabetes in England, 2009–2019
Source: Diabetes Obes Metab. 2025 Sep 1;27(11):6341–52. doi: 10.1111/dom.70025 (PMC12515765; doi:10.1111/dom.70025)
Supplement: Supplementary file 1 — Data S1. Supporting information. [file DOM-27-6341-s001.docx]

# Trends in mortality and hospitalisations for cardiovascular, kidney and liver disease in people with type 2 diabetes in England, 2009-2019

**Supplemental Material Content Page**

| Supplementary Table S1: Cohorts of people with type 2 diabetes included in each year of analysis | 2 |
| --- | --- |
| Supplementary Table S2: General Practice Participation in the National Diabetes Audit | 3 |
| Supplementary Table S3: Censoring in analysis of hospitalisations | 3 |
| Supplementary Table S4a: Rate ratios for all cause and cause specific mortality for people aged 20-74 years | 4 |
| Supplementary Table S4b: Rate ratios for all cause and cause specific mortality for people aged 75 years and older | 5 |
| Supplementary Table S5: Mortality rates per 1000 person-years by cause adjusted for age, sex, ethnicity, socio-economic deprivation and duration of diabetes by time period | 6 |
| Supplementary Table S6: Rate ratios for the time period 2018 to 2019 compared to the reference time period by ethnicity for each age group | 7 |
| Supplementary Table S7: Rate ratios for the time period 2018 to 2019 compared to the reference time period by socio-economic deprivation for each age group | 8 |
| Supplementary Table S8: Rate ratios for the time period 2018 to 2019 compared to the reference time period by duration of diagnosed diabetes for each age group | 9 |
| Supplementary Table S9: Rate ratios for the time period 2018 to 2019 compared to the reference time period by sex for each age group | 9 |
| Supplementary Figure 1: Cumulative proportion of mortality by cause adjusted for age, sex, ethnicity, deprivation and duration of diagnosed diabetes for people aged 20-74 years and aged 75 years and older | 10 |
| Supplementary Table S10: Top 10 ICD-10 codes for underlying cause of death by age group and time period | 11 |
| Additional information on data sources | 12 |
| Additional information on statistical methods | 12 |
| Explanation of the need for a 21 month period between end of the audit period and start of active follow-up in this analysis | 13 |
| Information Governance | 13 |

Supplemental Table S1: Cohorts of people with type 2 diabetes included in each year of analysis

|  | | NDA data collection used to identify cohort | Number of people with type 2 diabetes included in the NDA data collection | Start of follow up period | Number alive at start of follow up period | End of follow up period | Total person years of follow up in analysis of mortality |
| --- | --- | --- | --- | --- | --- | --- | --- |
| 2009-2011 | 2009 | 2006/07 | 1,057,365 | 01/01/2009 | 1,010,535 | 31/12/2009 | 991,228 |
|  | 2010 | 2007/08 | 1,240,585 | 01/01/2010 | 1,172,500 | 31/12/2010 | 1,150,330 |
|  | 2011 | 2008/09 | 1,502,575 | 01/01/2011 | 1,379,455 | 31/12/2011 | 1,353,763 |
| 2012-2014 | 2012 | 2009/10 | 1,764,580 | 01/01/2012 | 1,613,190 | 31/12/2012 | 1,582,368 |
|  | 2013 | 2010/11 | 1,981,475 | 01/01/2013 | 1,802,945 | 31/12/2013 | 1,767,936 |
|  | 2014 | 2011/12 | 2,206,935 | 01/01/2014 | 1,975,525 | 31/12/2014 | 1,939,592 |
| 2015-2017 | 2015 | 2012/13 | 1,849,035 | 01/01/2015 | 1,661,215 | 31/12/2015 | 1,627,868 |
|  | 2016 | 2013/14 | 1,570,965 | 01/01/2016 | 1,384,990 | 31/12/2016 | 1,357,524 |
|  | 2017 | 2014/15 | 1,691,205 | 01/01/2017 | 1,440,870 | 31/12/2017 | 1,411,930 |
| 2018-2019 | 2018 | 2015/16 | 2,445,820 | 01/01/2018 | 2,135,700 | 31/12/2018 | 2,092,481 |
|  | 2019 | 2016/17 | 2,875,220 | 01/01/2019 | 2,535,630 | 31/12/2019 | 2,487,125 |

Supplementary Table S2: General Practice participation in the National Diabetes Audit

| Audit period | Time period for events | Number of general practices included in the NDA | % of general practices included in the NDA |
| --- | --- | --- | --- |
| 2006/07 | 2009 | 4900 | 48.1% |
| 2007/08 | 2010 | 5359 | 64.6% |
| 2008/09 | 2011 | 5920 | 70.8% |
| 2009/10 | 2012 | 6507 | 77.9% |
| 2010/11 | 2013 | 6791 | 83.0% |
| 2011/12 | 2014 | 7140 | 88.4% |
| 2012/13 | 2015 | 5666 | 70.8% |
| 2013/14 | 2016 | 4274 | 54.9% |
| 2014/15 | 2017 | 4256 | 54.9% |
| 2015/16 | 2018 | 6165 | 81.4% |
| 2016/17 | 2019 | 6942 | 95.0% |

Supplemental Table S3: Censoring in analysis of hospitalisations

|  | Number (%) of records censored |
| --- | --- |
| Myocardial infarction | 661,285 (3.7%) |
| Stroke | 654,333 (3.6%) |
| Heart failure | 638,265 (3.5%) |
| Kidney disease | 641,481 (3.5%) |
| Liver disease | 641,480 (3.5%) |

Supplemental Table S4a: Rate ratios for all cause and cause specific mortality for people aged 20-74 years

|  | | All-cause | Cardiovascular disease | Cancer | Kidney disease | Liver disease | Respiratory disease | Infections | Dementia | Diabetes |
| --- | --- | --- | --- | --- | --- | --- | --- | --- | --- | --- |
|  |  | RR (95% CI) | RR (95% CI) | RR (95% CI) | RR (95% CI) | RR (95% CI) | RR (95% CI) | RR (95% CI) | RR (95% CI) | RR (95% CI) |
| Time period | |  |  |  |  |  |  |  |  |  |
|  | 2009-11 | 1.00 | 1.00 | 1.00 | 1.00 | 1.00 | 1.00 | 1.00 | 1.00 | 1.00 |
|  | 2012-14 | 0.97 (0.96-0.98) | 0.90 (0.88-0.92) | 0.98 (0.96-1.01) | 0.92 (0.80-1.06) | 1.00 (0.94-1.07) | 1.08 (1.04-1.12) | 1.00 (0.89-1.12) | 1.68 (1.49-1.89) | 0.92 (0.87-0.99) |
|  | 2015-17 | 0.99 (0.98-1.01) | 0.90 (0.88-0.92) | 0.98 (0.96-1.00) | 0.82 (0.71-0.95) | 1.11 (1.04-1.18) | 1.17 (1.13-1.22) | 1.04 (0.93-1.17) | 2.26 (2.01-2.54) | 0.97 (0.91-1.04) |
|  | 2018-19 | 0.96 (0.95-0.97) | 0.82 (0.80-0.84) | 0.93 (0.91-0.96) | 0.78 (0.67-0.91) | 1.01 (0.95-1.08) | 1.17 (1.13-1.22) | 0.88 (0.78-0.99) | 2.26 (2.01-2.54) | 1.01 (0.95-1.08) |
| Sex |  |  |  |  |  |  |  |  |  |  |
|  | Female | 0.82 (0.81-0.82) | 0.64 (0.63-0.65) | 0.85 (0.84-0.87) | 1.15 (1.04-1.27) | 0.74 (0.71-0.78) | 1.02 (0.99-1.05) | 1.03 (0.95-1.12) | 1.10 (1.03-1.18) | 0.91 (0.87-0.95) |
|  | Male | 1.00 | 1.00 | 1.00 | 1.00 | 1.00 | 1.00 | 1.00 | 1.00 | 1.00 |
| Age |  |  |  |  |  |  |  |  |  |  |
|  | Per additional year | 1.08 (1.08-1.08) | 1.08 (1.08-1.08) | 1.09 (1.09-1.09) | 1.08 (1.08-1.09) | 1.01 (1.01-1.02) | 1.11 (1.11-1.11) | 1.06 (1.06-1.07) | 1.28 (1.27-1.29) | 1.06 (1.06-1.07) |
| Deprivation | |  |  |  |  |  |  |  |  |  |
|  | Most deprived | 1.00 | 1.00 | 1.00 | 1.00 | 1.00 | 1.00 | 1.00 | 1.00 | 1.00 |
|  | 2nd most deprived | 0.84 (0.83-0.85) | 0.82 (0.80-0.84) | 0.88 (0.86-0.90) | 0.81 (0.71-0.92) | 0.85 (0.80-0.90) | 0.78 (0.76-0.81) | 0.83 (0.75-0.93) | 0.86 (0.78-0.95) | 0.80 (0.76-0.85) |
|  | 3rd most deprived | 0.71 (0.70-0.72) | 0.67 (0.66-0.69) | 0.81 (0.80-0.83) | 0.61 (0.53-0.71) | 0.74 (0.69-0.79) | 0.58 (0.56-0.60) | 0.65 (0.57-0.73) | 0.76 (0.69-0.84) | 0.65 (0.61-0.70) |
|  | 2nd least deprived | 0.63 (0.62-0.64) | 0.59 (0.58-0.61) | 0.75 (0.73-0.77) | 0.48 (0.41-0.56) | 0.67 (0.62-0.72) | 0.47 (0.45-0.49) | 0.60 (0.53-0.68) | 0.67 (0.60-0.74) | 0.53 (0.49-0.57) |
|  | Least deprived | 0.55 (0.54-0.56) | 0.50 (0.49-0.52) | 0.68 (0.67-0.70) | 0.39 (0.32-0.47) | 0.60 (0.56-0.65) | 0.38 (0.36-0.39) | 0.56 (0.49-0.64) | 0.61 (0.54-0.68) | 0.44 (0.41-0.48) |
|  | Missing | 0.81 (0.76-0.86) | 0.80 (0.72-0.88) | 0.82 (0.74-0.92) | 1.21 (0.71-2.06) | 0.95 (0.72-1.24) | 0.67 (0.56-0.80) | 0.70 (0.40-1.21) | 0.83 (0.53-1.29) | 0.78 (0.58-1.03) |
| Ethnicity | |  |  |  |  |  |  |  |  |  |
|  | White | 1.00 | 1.00 | 1.00 | 1.00 | 1.00 | 1.00 | 1.00 | 1.00 | 1.00 |
|  | Mixed | 0.59 (0.56-0.62) | 0.68 (0.62-0.75) | 0.54 (0.49-0.60) | 0.37 (0.16-0.82) | 0.43 (0.32-0.58) | 0.42 (0.34-0.51) | 0.72 (0.45-1.15) | 0.68 (0.44-1.05) | 0.89 (0.71-1.12) |
|  | South Asian | 0.53 (0.52-0.54) | 0.69 (0.67-0.71) | 0.42 (0.40-0.43) | 0.73 (0.61-0.87) | 0.34 (0.31-0.38) | 0.45 (0.42-0.47) | 1.03 (0.91-1.17) | 0.47 (0.40-0.55) | 0.75 (0.69-0.81) |
|  | Black | 0.52 (0.50-0.53) | 0.58 (0.55-0.61) | 0.53 (0.51-0.56) | 0.55 (0.41-0.74) | 0.20 (0.16-0.24) | 0.31 (0.28-0.34) | 0.75 (0.61-0.93) | 0.62 (0.51-0.76) | 0.78 (0.69-0.87) |
|  | Other | 0.65 (0.63-0.67) | 0.72 (0.69-0.75) | 0.65 (0.62-0.68) | 0.61 (0.45-0.82) | 0.48 (0.42-0.55) | 0.56 (0.52-0.61) | 0.90 (0.74-1.1) | 0.74 (0.61-0.90) | 0.73 (0.65-0.83) |
|  | Missing | 0.63 (0.61-0.65) | 1.12 (1.07-1.18) | 0.36 (0.33-0.39) | 0.20 (0.09-0.44) | 0.26 (0.20-0.35) | 0.43 (0.37-0.49) | 0.39 (0.25-0.61) | 0.35 (0.23-0.52) | 0.47 (0.38-0.59) |
| Duration of diagnosis | |  |  |  |  |  |  |  |  |  |
|  | Per additional year | 1.01 (1.01-1.01) | 1.01 (1.01-1.01) | 1.01 (1.01-1.01) | 1.01 (1.01-1.01) | 1.01 (1.01-1.01) | 1.01 (1.01-1.01) | 1.01 (1.01-1.01) | 1.01 (1.01-1.01) | 1.02 (1.01-1.02) |

Supplemental Table S4b: Rate ratios for all cause and cause specific mortality for people aged 75 years and older

|  | | All-cause | Cardiovascular disease | Cancer | Kidney disease | Liver disease | Respiratory disease | Infections | Dementia | Diabetes |
| --- | --- | --- | --- | --- | --- | --- | --- | --- | --- | --- |
|  |  | RR (95% CI) | RR (95% CI) | RR (95% CI) | RR (95% CI) | RR (95% CI) | RR (95% CI) | RR (95% CI) | RR (95% CI) | RR (95% CI) |
| Time period | |  |  |  |  |  |  |  |  |  |
|  | 2009-11 | 1.00 | 1.00 | 1.00 | 1.00 | 1.00 | 1.00 | 1.00 | 1.00 | 1.00 |
|  | 2012-14 | 0.99 (0.98-1.00) | 0.88 (0.87-0.89) | 1.02 (1.00-1.04) | 0.86 (0.79-0.93) | 1.15 (1.04-1.27) | 1.01 (0.98-1.03) | 0.99 (0.92-1.08) | 1.71 (1.65-1.76) | 0.84 (0.81-0.87) |
|  | 2015-17 | 1.02 (1.01-1.02) | 0.83 (0.82-0.84) | 1.03 (1.01-1.04) | 0.75 (0.69-0.82) | 1.22 (1.10-1.36) | 1.02 (0.99-1.04) | 1.10 (1.01-1.19) | 2.27 (2.20-2.34) | 0.85 (0.82-0.88) |
|  | 2018-19 | 0.98 (0.97-0.98) | 0.75 (0.73-0.76) | 1.00 (0.98-1.02) | 0.75 (0.69-0.82) | 1.23 (1.10-1.36) | 0.98 (0.96-1.01) | 0.97 (0.89-1.05) | 2.36 (2.29-2.43) | 0.78 (0.76-0.81) |
| Sex |  |  |  |  |  |  |  |  |  |  |
|  | Female | 0.80 (0.79-0.8) | 0.77 (0.76-0.78) | 0.64 (0.63-0.65) | 0.83 (0.78-0.88) | 0.85 (0.79-0.91) | 0.73 (0.72-0.74) | 1.01 (0.96-1.07) | 1.13 (1.11-1.15) | 0.94 (0.92-0.97) |
|  | Male | 1.00 | 1.00 | 1.00 | 1.00 | 1.00 | 1.00 | 1.00 | 1.00 | 1.00 |
| Age | |  |  |  |  |  |  |  |  |  |
|  | Per additional year | 1.11 (1.11-1.11) | 1.11 (1.1-1.11) | 1.05 (1.05-1.05) | 1.12 (1.11-1.12) | 0.96 (0.96-0.97) | 1.13 (1.13-1.13) | 1.10 (1.09-1.11) | 1.17 (1.17-1.17) | 1.15 (1.15-1.15) |
| Deprivation | |  |  |  |  |  |  |  |  |  |
|  | Most deprived | 1.00 | 1.00 | 1.00 | 1.00 | 1.00 | 1.00 | 1.00 | 1.00 | 1.00 |
|  | 2nd most deprived | 0.93 (0.92-0.93) | 0.95 (0.93-0.96) | 0.93 (0.92-0.95) | 0.94 (0.86-1.03) | 0.88 (0.79-0.98) | 0.85 (0.83-0.87) | 0.87 (0.80-0.94) | 0.93 (0.90-0.95) | 0.93 (0.90-0.97) |
|  | 3rd most deprived | 0.86 (0.86-0.87) | 0.89 (0.88-0.91) | 0.86 (0.85-0.88) | 0.87 (0.80-0.95) | 0.88 (0.79-0.98) | 0.76 (0.74-0.77) | 0.82 (0.76-0.89) | 0.87 (0.85-0.89) | 0.91 (0.88-0.95) |
|  | 2nd least deprived | 0.82 (0.81-0.83) | 0.85 (0.84-0.87) | 0.83 (0.81-0.84) | 0.79 (0.72-0.87) | 0.83 (0.75-0.93) | 0.69 (0.68-0.71) | 0.75 (0.69-0.82) | 0.84 (0.82-0.86) | 0.87 (0.83-0.90) |
|  | Least deprived | 0.77 (0.77-0.78) | 0.78 (0.77-0.80) | 0.80 (0.78-0.82) | 0.64 (0.58-0.71) | 0.75 (0.67-0.84) | 0.64 (0.62-0.66) | 0.69 (0.63-0.75) | 0.81 (0.79-0.83) | 0.83 (0.79-0.86) |
|  | Missing | 0.86 (0.83-0.90) | 0.83 (0.77-0.90) | 0.89 (0.81-0.97) | 0.75 (0.48-1.17) | 0.77 (0.46-1.28) | 0.77 (0.70-0.86) | 0.82 (0.57-1.18) | 0.84 (0.75-0.94) | 1.02 (0.87-1.20) |
| Ethnicity | |  |  |  |  |  |  |  |  |  |
|  | White | 1.00 | 1.00 | 1.00 | 1.00 | 1.00 | 1.00 | 1.00 | 1.00 | 1.00 |
|  | Mixed | 0.69 (0.66-0.72) | 0.76 (0.71-0.82) | 0.64 (0.58-0.7) | 0.80 (0.53-1.22) | 0.62 (0.37-1.03) | 0.53 (0.46-0.6) | 0.90 (0.63-1.28) | 0.75 (0.66-0.85) | 0.99 (0.84-1.17) |
|  | South Asian | 0.67 (0.66-0.68) | 0.84 (0.82-0.86) | 0.44 (0.42-0.46) | 1.04 (0.91-1.18) | 0.72 (0.61-0.85) | 0.65 (0.62-0.68) | 1.19 (1.06-1.33) | 0.53 (0.50-0.56) | 0.92 (0.87-0.98) |
|  | Black | 0.62 (0.61-0.63) | 0.67 (0.65-0.70) | 0.62 (0.60-0.65) | 0.79 (0.66-0.94) | 0.35 (0.27-0.47) | 0.37 (0.35-0.40) | 0.55 (0.45-0.66) | 0.70 (0.67-0.74) | 0.96 (0.90-1.03) |
|  | Other | 0.89 (0.87-0.90) | 0.96 (0.93-0.99) | 0.83 (0.79-0.86) | 1.24 (1.05-1.45) | 0.73 (0.58-0.92) | 0.84 (0.8-0.88) | 1.08 (0.93-1.27) | 0.82 (0.78-0.87) | 1.06 (0.99-1.14) |
|  | Missing | 0.79 (0.77-0.81) | 1.05 (1.00-1.09) | 0.65 (0.61-0.70) | 0.73 (0.54-0.99) | 0.69 (0.49-0.98) | 0.65 (0.60-0.70) | 0.65 (0.49-0.87) | 0.59 (0.54-0.65) | 0.78 (0.69-0.88) |
| Duration of diagnosis (years) | |  |  |  |  |  |  |  |  |  |
|  | Per additional year | 1.01 (1.01-1.01) | 1.01 (1.01-1.01) | 1.00 (1.00-1.00) | 1.01 (1.01-1.01) | 1.00 (1.00-1.01) | 1.01 (1.00-1.01) | 1.01 (1.01-1.01) | 1.01 (1.01-1.01) | 1.01 (1.01-1.01) |

Supplemental Table S5: Mortality rates per 1000 person-years and rate ratios by cause adjusted for age, sex, ethnicity, socio-economic deprivation and duration of diabetes by time period

|  | | 2009-11 | | 2012-14 | | 2015-17 | | 2018-19 | |
| --- | --- | --- | --- | --- | --- | --- | --- | --- | --- |
|  |  | Rate per 1000 person-years | RR (reference time period) | Rate per 1000 person-years | RR (95% CI) | Rate per 1000 person-years | RR (95% CI) | Rate per 1000 person-years | RR (95% CI) |
| Age 20-74 years | All-cause | 16.6 | 1.00 | 16.1 | 0.97 (0.96-0.98) | 16.5 | 0.99 (0.98-1.01) | 15.9 | 0.96 (0.95-0.97) |
|  | CVD | 5.9 | 1.00 | 5.3 | 0.90 (0.88-0.92) | 5.3 | 0.90 (0.88-0.92) | 4.8 | 0.82 (0.80-0.84) |
|  | Cancer | 5.5 | 1.00 | 5.4 | 0.98 (0.96-1.01) | 5.3 | 0.98 (0.96-1.00) | 5.1 | 0.93 (0.91-0.96) |
|  | Renal disease | 0.1 | 1.00 | 0.1 | 0.92 (0.80-1.06) | 0.1 | 0.82 (0.71-0.95) | 0.1 | 0.78 (0.67-0.91) |
|  | Liver disease | 0.6 | 1.00 | 0.6 | 1.00 (0.94-1.07) | 0.7 | 1.11 (1.04-1.18) | 0.6 | 1.01 (0.95-1.08) |
|  | Respiratory disease | 1.6 | 1.00 | 1.7 | 1.08 (1.04-1.12) | 1.9 | 1.17 (1.13-1.22) | 1.9 | 1.17 (1.13-1.22) |
|  | Infections | 0.2 | 1.00 | 0.2 | 1.00 (0.89-1.12) | 0.2 | 1.04 (0.93-1.17) | 0.2 | 0.88 (0.78-0.99) |
|  | Dementia | 0.15 | 1.00 | 0.25 | 1.68 (1.49-1.89) | 0.34 | 2.26 (1.06-2.01) | 0.34 | 2.26 (1.06-2.01) |
|  | Diabetes | 0.7 | 1.00 | 0.6 | 0.92 (0.87-0.99) | 0.6 | 0.97 (0.91-1.04) | 0.7 | 1.01 (0.95-1.08) |
|  | Other causes | 1.9 |  | 1.9 |  | 2.1 |  | 2.3 |  |
| Age 75+ years | All-cause | 93.4 | 1.00 | 92.3 | 0.99 (0.98-1.00) | 94.8 | 1.02 (1.01-1.02) | 91.1 | 0.98 (0.97-0.98) |
|  | CVD | 33.5 | 1.00 | 29.5 | 0.88 (0.87-0.89) | 27.8 | 0.83 (0.82-0.84) | 25.0 | 0.75 (0.73-0.76) |
|  | Cancer | 15.8 | 1.00 | 16.2 | 1.02 (1.00-1.04) | 16.2 | 1.03 (1.01-1.04) | 15.8 | 1.00 (0.98-1.02) |
|  | Renal disease | 1.1 | 1.00 | 0.9 | 0.86 (0.79-0.93) | 0.8 | 0.75 (0.69-0.82) | 0.8 | 0.75 (0.69-0.82) |
|  | Liver disease | 0.5 | 1.00 | 0.6 | 1.15 (1.04-1.27) | 0.6 | 1.22 (1.04-1.36) | 0.6 | 1.23 (1.10-1.36) |
|  | Respiratory disease | 13.4 | 1.00 | 13.5 | 1.01 (0.98-1.03) | 13.6 | 1.02 (0.99-1.04) | 13.2 | 0.98 (0.96-1.01) |
|  | Infections | 1.2 | 1.00 | 1.2 | 0.99 (0.92-1.08) | 1.3 | 1.10 (1.01-1.19) | 1.1 | 0.97 (0.89-1.05) |
|  | Dementia | 7.3 | 1.00 | 12.5 | 1.71 (1.65-1.76) | 16.7 | 2.27 (2.20-2.34) | 17.3 | 2.36 (2.29-2.43) |
|  | Diabetes | 0.0 | 1.00 | 0.0 | 0.84 (0.81-0.87) | 0.0 | 0.85 (0.82-0.88) | 0.0 | 0.78 (0.76-0.81) |
|  | Other causes | 20.5 |  | 17.9 |  | 17.7 |  | 17.2 |  |

Supplemental Table S6: Rate ratios for the time period 2018 to 2019 compared to the reference time period by ethnicity for each age group

|  | | All-cause mortality | Hospitalisation for myocardial infarction | Hospitalisation for stroke | Hospitalisation for heart failure | Composite CVD indicator | Hospitalisation for kidney disease | Hospitalisation for liver disease |
| --- | --- | --- | --- | --- | --- | --- | --- | --- |
|  |  | RR (95% CI ) | RR (95% CI ) | RR (95% CI ) | RR (95% CI ) | RR (95% CI ) | RR (95% CI ) | RR (95% CI ) |
| Age 20-74 years | White | 1.00 (0.99-1.01) | 1.21 (1.18-1.25) | 1.05 (1.02-1.08) | 1.13 (1.1-1.16) | 1.07 (1.05-1.08) | 1.15 (1.13-1.16) | 1.31 (1.26-1.261) |
|  | Mixed | 0.88 (0.75-1.04) | 1.87 (1.40-2.50) | 0.79 (0.60-1.03) | 0.92 (0.72-1.17) | 1.05 (0.91-1.20) | 1.12 (0.99-1.28) | 0.94 (0.63-1.38) |
|  | South Asian | 0.92 (0.87-0.96) | 1.15 (1.09-1.22) | 1.08 (1.00-1.17) | 1.10 (1.02-1.18) | 1.06 (1.03-1.10) | 1.08 (1.04-1.13) | 1.13 (1.01-1.26) |
|  | Black | 1.05 (0.97-1.13) | 1.28 (1.10-1.49) | 1.18 (1.05-1.33) | 1.33 (1.18-1.49) | 1.18 (1.11-1.26) | 1.20 (1.13-1.27) | 1.26 (1.01-1.58) |
|  | Other | 0.74 (0.68-0.80) | 1.07 (0.95-1.20) | 0.98 (0.86-1.12) | 1.02 (0.90-1.16) | 0.93 (0.87-1.00) | 1.02 (0.96-1.09) | 1.10 (0.94-1.30) |
|  | Missing | 0.17 (0.15-0.18) | 1.02 (0.64-1.61) | 0.76 (0.50-1.16) | 0.88 (0.51-1.51) | 0.22 (0.19-0.25) | 2.29 (1.64-3.20) | 0.71 (0.41-1.22) |
|  | p value | <.0001 | <.0001 | 0.001 | <.0001 | <.0001 | <.0001 | <.0001 |
| Age ≥75 years | White | 1.00 (0.99-1.01) | 0.84 (0.82-0.86) | 0.91 (0.89-0.93) | 1.15 (1.13-1.18) | 0.91 (0.90-0.92) | 1.09 (1.07-1.11) | 1.56 (1.45-1.67) |
|  | Mixed | 0.95 (0.83-1.08) | 0.74 (0.52-1.06) | 0.79 (0.60-1.03) | 1.32 (1.04-1.69) | 0.91 (0.79-1.05) | 1.07 (0.89-1.29) | 0.61 (0.20-1.90) |
|  | South Asian | 0.99 (0.94-1.04) | 0.98 (0.90-1.07) | 0.94 (0.85-1.04) | 1.27 (1.18-1.37) | 0.98 (0.94-1.03) | 1.05 (0.98-1.12) | 1.19 (0.89-1.58) |
|  | Black | 1.03 (0.97-1.10) | 1.10 (0.93-1.31) | 0.97 (0.86-1.10) | 1.29 (1.16-1.43) | 1.05 (0.99-1.12) | 1.21 (1.12-1.31) | 1.46 (0.92-2.31) |
|  | Other | 0.87 (0.82-0.92) | 0.89 (0.77-1.02) | 0.86 (0.75-0.99) | 1.19 (1.06-1.33) | 0.91 (0.85-0.97) | 0.98 (0.89-1.08) | 0.92 (0.65-1.32) |
|  | Missing | 0.22 (0.20-0.24) | 0.34 (0.22-0.51) | 0.43 (0.32-0.59) | 0.51 (0.36-0.72) | 0.23 (0.20-0.26) | 1.21 (0.85-1.73) | 0.74 (0.30-1.83) |
|  | p value | <.0001 | <.0001 | <.0001 | <.0001 | <.0001 | <.0001 | <.0001 |
| Models adjusted for age, sex, social deprivation, duration of diagnosed diabetes, ethnicity, deprivation quintile and time period*ethnicity | | | | | | | |  |
| P -value reported is the p-value for the inclusion of the interaction term within the Poisson regression | | | | | | | |  |

Supplemental Table S7: Rate ratios for the time period 2018 to 2019 compared to reference time period by socio-economic deprivation for each age group

|  | | All-cause mortality | Hospitalisation for myocardial infarction | Hospitalisation for stroke | Hospitalisation for heart failure | Composite CVD indicator | Hospitalisation for kidney disease | Hospitalisation for liver disease |
| --- | --- | --- | --- | --- | --- | --- | --- | --- |
|  |  | RR (95% CI ) | RR (95% CI ) | RR (95% CI ) | RR (95% CI ) | RR (95% CI ) | RR (95% CI ) | RR (95% CI ) |
| Age 20-74 years | Most deprived | 0.97 (0.95-1.00) | 1.14 (1.10-1.19) | 1.04 (1.00-1.10) | 1.09 (1.05-1.14) | 1.04 (1.02-1.06) | 1.13 (1.11-1.16) | 1.17 (1.10-1.24) |
|  | 2nd most deprived | 0.97 (0.94-1.00) | 1.16 (1.10-1.22) | 1.01 (0.95-1.06) | 1.13 (1.08-1.19) | 1.02 (1.00-1.05) | 1.11 (1.08-1.14) | 1.28 (1.19-1.37) |
|  | 3rd most deprived | 0.96 (0.93-0.99) | 1.29 (1.22-1.36) | 1.10 (1.03-1.17) | 1.17 (1.11-1.23) | 1.09 (1.06-1.12) | 1.14 (1.11-1.18) | 1.34 (1.24-1.44) |
|  | 2nd least deprived | 0.95 (0.92-0.98) | 1.21 (1.14-1.29) | 1.03 (0.97-1.10) | 1.15 (1.08-1.22) | 1.04 (1.01-1.08) | 1.15 (1.12-1.2) | 1.33 (1.23-1.44) |
|  | Least deprived | 0.91 (0.88-0.94) | 1.30 (1.21-1.39) | 1.09 (1.01-1.18) | 1.10 (1.02-1.18) | 1.04 (1.00-1.08) | 1.14 (1.10-1.19) | 1.37 (1.25-1.50) |
|  | p value | <.0001 | <.0001 | 0.002 | <.0001 | <.0001 | <.0001 | <.0001 |
| Age ≥75 years | Most deprived | 1.00 (0.98-1.02) | 0.83 (0.79-0.88) | 0.88 (0.84-0.93) | 1.14 (1.10-1.19) | 0.90 (0.88-0.92) | 1.10 (1.07-1.14) | 1.36 (1.17-1.57) |
|  | 2nd most deprived | 0.98 (0.96-1.00) | 0.85 (0.81-0.90) | 0.95 (0.90-0.99) | 1.13 (1.09-1.18) | 0.90 (0.88-0.92) | 1.06 (1.02-1.10) | 1.34 (1.16-1.56) |
|  | 3rd most deprived | 0.97 (0.95-0.98) | 0.85 (0.81-0.89) | 0.87 (0.83-0.91) | 1.16 (1.11-1.20) | 0.89 (0.88-0.91) | 1.12 (1.08-1.16) | 1.53 (1.31-1.78) |
|  | 2nd least deprived | 0.96 (0.95-0.98) | 0.86 (0.82-0.91) | 0.89 (0.85-0.94) | 1.19 (1.14-1.24) | 0.89 (0.87-0.92) | 1.07 (1.03-1.11) | 1.61 (1.38-1.87) |
|  | Least deprived | 0.97 (0.95-0.99) | 0.88 (0.83-0.93) | 0.97 (0.92-1.02) | 1.19 (1.14-1.25) | 0.92 (0.89-0.94) | 1.09 (1.04-1.13) | 1.72 (1.45-2.05) |
|  | p value | <.0001 | <.0001 | <.0001 | <.0001 | <.0001 | <.0001 | <.0001 |
| Models adjusted for age, sex, ethnicity, duration of diagnosed diabetes, ethnicity, deprivation quintile and time period*deprivation quintile | | | | | | | | |
| P -value reported is the p-value for the inclusion of the interaction term within the Poisson regression | | | | | | | | |

Supplemental Table S8: Rate ratios for the time period 2018 to 2019 compared to reference time period by duration of diagnosed diabetes for each age group

|  | | All-cause mortality | Hospitalisation for myocardial infarction | Hospitalisation for stroke | Hospitalisation for heart failure | Composite CVD indicator | Hospitalisation for kidney disease | Hospitalisation for liver disease |
| --- | --- | --- | --- | --- | --- | --- | --- | --- |
|  |  | RR (95% CI ) | RR (95% CI ) | RR (95% CI ) | RR (95% CI ) | RR (95% CI ) | RR (95% CI ) | RR (95% CI ) |
| Age 20-74 years | <5 years | 0.93 (0.91-0.96) | 1.25 (1.19-1.32) | 1.03 (0.98-1.09) | 1.14 (1.08-1.21) | 1.06 (1.03-1.09) | 1.13 (1.10-1.17) | 0.47 (0.11-1.94) |
|  | 5-9·9 years | 0.96 (0.93-0.98) | 1.27 (1.21-1.33) | 1.12 (1.06-1.18) | 1.19 (1.13-1.25) | 1.10 (1.07-1.13) | 1.21 (1.17-1.24) | 1.12 (1.06-1.19) |
|  | 10-14.9 years | 1.03 (1.00-1.06) | 1.44 (1.37-1.52) | 1.33 (1.26-1.41) | 1.57 (1.49-1.65) | 1.31 (1.28-1.35) | 1.42 (1.38-1.46) | 1.31 (1.23-1.39) |
|  | ≥15 years | 1.29 (1.25-1.33) | 1.84 (1.74-1.94) | 1.66 (1.56-1.77) | 2.34 (2.22-2.47) | 1.75 (1.70-1.80) | 2.01 (1.94-2.08) | 1.42 (1.33-1.52) |
|  | p value | <.0001 | <.0001 | <.0001 | <.0001 | <.0001 | <.0001 | <.0001 |
| Age ≥75 years | <5 years | 1.12 (0.94-1.34) | 0.90 (0.85-0.96) | 0.91 (0.86-0.96) | 1.14 (1.09-1.20) | 0.91 (0.88-0.93) | 1.10 (1.05-1.15) | 1.04 (0.87-1.24) |
|  | 5-9·9 years | 0.97 (0.95-0.99) | 0.90 (0.85-0.96) | 0.90 (0.86-0.94) | 1.17 (1.12-1.22) | 0.90 (0.88-0.92) | 1.20 (1.15-1.25) | 1.53 (1.32-1.78) |
|  | 10-14.9 years | 0.98 (0.96-1.00) | 0.83 (0.78-0.87) | 0.96 (0.92-1.01) | 1.25 (1.20-1.30) | 0.97 (0.95-0.99) | 1.43 (1.37-1.48) | 1.91 (1.64-2.21) |
|  | ≥15 years | 1.08 (1.06-1.10) | 0.93 (0.88-0.98) | 1.09 (1.03-1.14) | 1.54 (1.47-1.61) | 1.15 (1.12-1.18) | 1.79 (1.72-1.87) | 2.08 (1.76-2.46) |
|  | p value | <.0001 | <.0001 | <.0001 | <.0001 | <.0001 | <.0001 | <.0001 |
| Models adjusted for age, sex, ethnicity, duration of diabetes, ethnicity quintile and time period*duration of diabetes  P -value reported is the p-value for the inclusion of the interaction term within the Poisson regression  P -value reported is the p-value for the inclusion of the interaction term within the Poisson regression | | | | | | |  |  |
| P -value reported is the p-value for the inclusion of the interaction term within the Poisson regression | | | | | | |  |  |

Supplemental Table S9: Rate ratios for the time period 2018 to 2019 compared to reference time period by sex for each age group

|  | | All-cause mortality | Hospitalisation for myocardial infarction | Hospitalisation for stroke | Hospitalisation for heart failure | Composite CVD indicator | Hospitalisation for kidney disease | Hospitalisation for liver disease |
| --- | --- | --- | --- | --- | --- | --- | --- | --- |
|  |  | RR (95% CI ) | RR (95% CI ) | RR (95% CI ) | RR (95% CI ) | RR (95% CI ) | RR (95% CI ) | RR (95% CI) |
| Age 20-74 years | Women | 0.94 (0.93-0.96) | 1.15 (1.10-1.20) | 1.01 (0.97-1.05) | 1.15 (1.11-1.20) | 1.03 (1.01-1.06) | 1.15 (1.13-1.18) | 1.32 (1.03-1.25) |
|  | Men | 0.99 (0.96-1.01) | 1.23 (1.19-1.26) | 1.08 (1.04-1.12) | 1.11 (1.08-1.15) | 1.06 (1.04-1.07) | 1.13 (1.11-1.15) | 1.25 (1.02-1.20) |
|  | p value | <.0001 | <.0001 | 0.0002 | <.0001 | <.0001 | <.0001 | <.0001 |
| Age ≥75 years | Women | 0.97 (0.96-0.98) | 0.81 (0.78-0.84) | 0.89 (0.86-0.92) | 1.17 (1.14-1.20) | 0.89 (0.87-0.90) | 1.08 (1.05-1.11) | 1.43 (1.05-1.29) |
|  | Men | 0.98 (0.97-0.99) | 0.89 (0.86-0.91) | 0.94 (0.91-0.97) | 1.16 (1.13-1.19) | 0.92 (0.9-0.93) | 1.10 (1.07-1.12) | 1.54 (1.05-1.41) |
|  | p value | <.0001 | <.0001 | <.0001 | <.0001 | <.0001 | <.0001 | <.0001 |
| Models adjusted for age, sex, ethnicity, duration of diabetes, ethnicity quintile and time period*duration of diabetes | | | | | | |  |  |
| P -value reported is the p-value for the inclusion of the interaction term within the Poisson regression | | | | | | |  |  |

Supplementary Figure 1: Cumulative proportion of mortality by cause adjusted for age, sex, ethnicity, deprivation and diabetes duration for people aged 20-74 years and aged 75 years and older

| 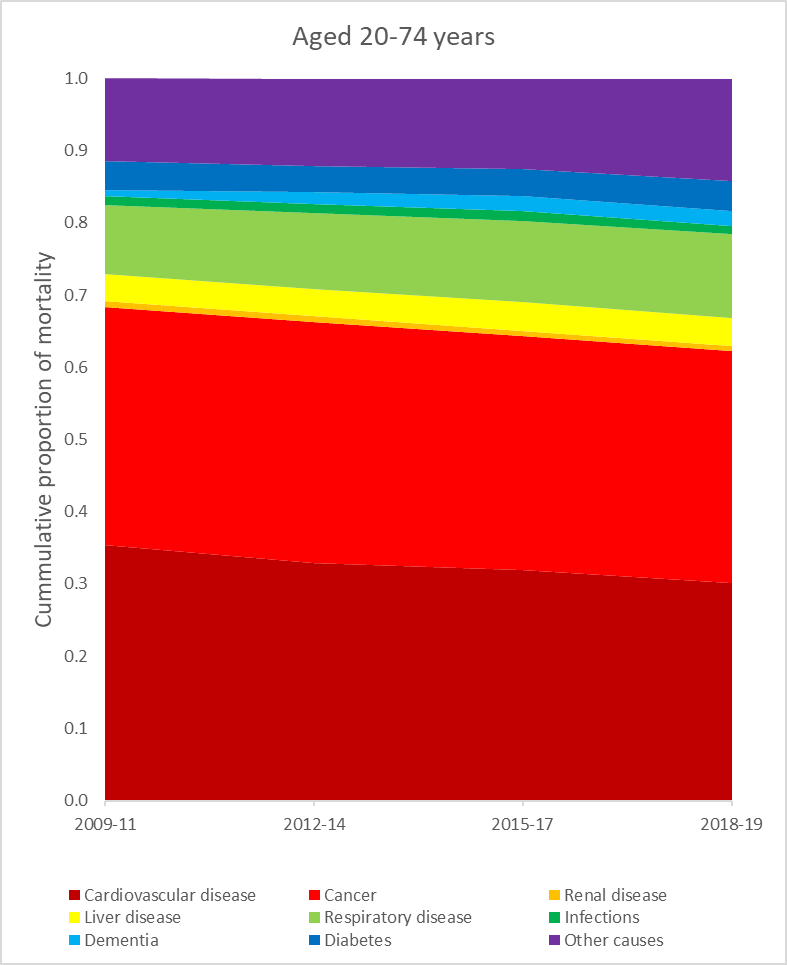 | 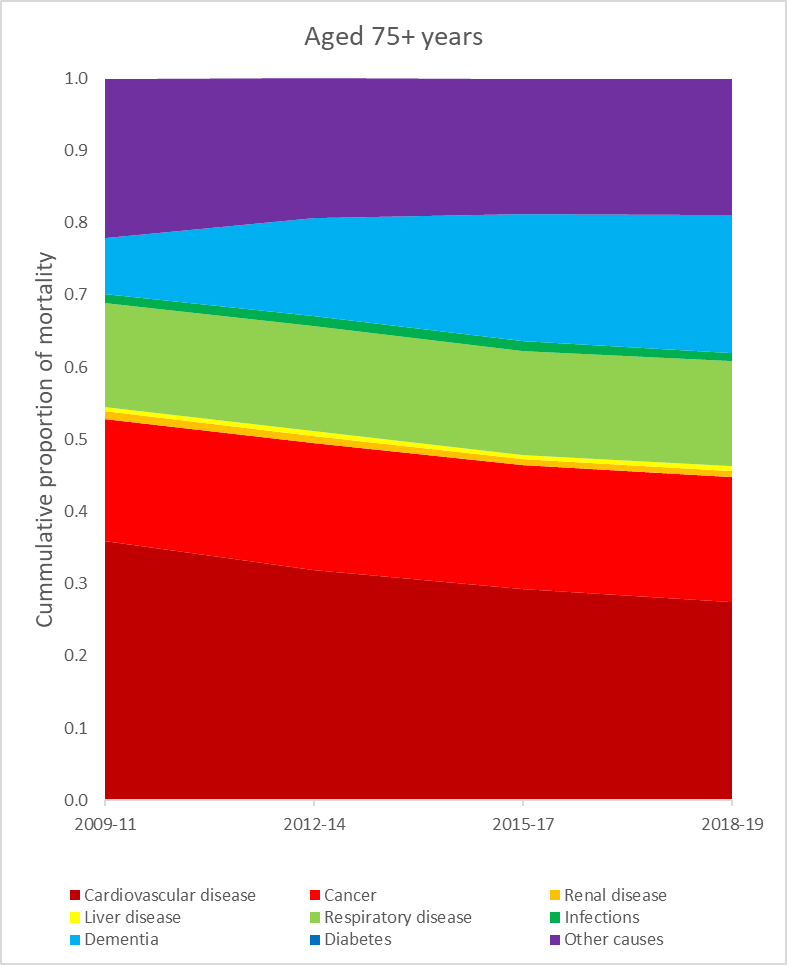 |
| --- | --- |

Supplementary Table S10: Top 10 ICD-10 codes for underlying cause of death by age group and time period

|  | Rank | 2009-11 | | 2012-2014 | | 2015-2017 | | 2018-2019 | |
| --- | --- | --- | --- | --- | --- | --- | --- | --- | --- |
|  |  | Code | Description | Code | Description | Code | Description | Code | Description |
| Age 20-74 years | 1 | I219 | Acute myocardial infarction | I219 | Acute myocardial infarction | I219 | Acute myocardial infarction | I219 | Acute myocardial infarction |
|  | 2 | I259 | Chronic ischaemic heart disease, unspecified | I259 | Chronic ischaemic heart disease, unspecified | I259 | Chronic ischaemic heart disease, unspecified | C349 | Malignant neoplasm of bronchus and lung |
|  | 3 | C349 | Malignant neoplasm of bronchus and lung | C349 | Malignant neoplasm of bronchus and lung | C349 | Malignant neoplasm of bronchus and lung | I259 | Chronic ischaemic heart disease, unspecified |
|  | 4 | I251 | Atherosclerotic heart disease | I251 | Atherosclerotic heart disease | I251 | Atherosclerotic heart disease | I251 | Atherosclerotic heart disease |
|  | 5 | I64 | Stroke, not specified as haemorrhage or infarction | C259 | Malignant neoplasm of pancreas | C259 | Malignant neoplasm of pancreas | C259 | Malignant neoplasm of pancreas |
|  | 6 | C259 | Malignant neoplasm of pancreas | J440 | Chronic obstructive pulmonary disease with acute lower respiratory infection | J440 | Chronic obstructive pulmonary disease with acute lower respiratory infection | J440 | Chronic obstructive pulmonary disease with acute lower respiratory infection |
|  | 7 | J449 | Chronic obstructive pulmonary disease, unspecified | I64 | Stroke, not specified as haemorrhage or infarction | J189 | Pneumonia, unspecified | J189 | Pneumonia, unspecified |
|  | 8 | C80 | Malignant neoplasm, without specification of site | C159 | Malignant neoplasm of oesophagus | I64 | Stroke, not specified as haemorrhage or infarction | I64 | Stroke, not specified as haemorrhage or infarction |
|  | 9 | C509 | Malignant neoplasm of breast | C509 | Malignant neoplasm of breast | C159 | Malignant neoplasm of oesophagus | J449 | Chronic obstructive pulmonary disease, unspecified |
|  | 10 | C159 | Malignant neoplasm of oesophagus | J189 | Pneumonia, unspecified | C509 | Malignant neoplasm of breast | C159 | Malignant neoplasm of oesophagus |
| Age 75+ years | 1 | I259 | Chronic ischaemic heart disease, unspecified | I259 | Chronic ischaemic heart disease, unspecified | F03 | Unspecified dementia | F03 | Unspecified dementia |
|  | 2 | I219 | Acute myocardial infarction | F03 | Unspecified dementia | I259 | Chronic ischaemic heart disease, unspecified | I259 | Chronic ischaemic heart disease, unspecified |
|  | 3 | I64 | Stroke, not specified as haemorrhage or infarction | I219 | Acute myocardial infarction | I219 | Acute myocardial infarction | I219 | Acute myocardial infarction |
|  | 4 | F03 | Unspecified dementia | I64 | Stroke, not specified as haemorrhage or infarction | J189 | Pneumonia, unspecified | J189 | Pneumonia, unspecified |
|  | 5 | C349 | Malignant neoplasm of bronchus and lung | C349 | Malignant neoplasm of bronchus and lung | I64 | Stroke, not specified as haemorrhage or infarction | C349 | Malignant neoplasm of bronchus and lung |
|  | 6 | I251 | Atherosclerotic heart disease | J189 | Pneumonia, unspecified | C349 | Malignant neoplasm of bronchus and lung | I64 | Stroke, not specified as haemorrhage or infarction |
|  | 7 | J180 | Bronchopneumonia, unspecified | I251 | Atherosclerotic heart disease | F019 | Vascular dementia, unspecified | G309 | Alzheimer disease |
|  | 8 | J189 | Pneumonia, unspecified | J180 | Bronchopneumonia, unspecified | J440 | Chronic obstructive pulmonary disease with acute lower respiratory infection | F019 | Vascular dementia, unspecified |
|  | 9 | J449 | Chronic obstructive pulmonary disease, unspecified | J440 | Chronic obstructive pulmonary disease with acute lower respiratory infection | G309 | Alzheimer disease | J440 | Chronic obstructive pulmonary disease with acute lower respiratory infection |
|  | 10 | E149 | Unspecified diabetes mellitus | F019 | Vascular dementia, unspecified | J180 | Bronchopneumonia, unspecified | C61 | Malignant neoplasm of prostate |

Additional information on data sources

The Indices of Multiple Deprivation use a combination of administrative and census data to create seven domains that are combined (income domain – 22.5%, employment domain – 22.5%, health deprivation and disability domain – 13.5%, education, skills and training domain - 13.5%, barriers to housing and services domain - 9.3%, crime domain - 9.3%, living environment domain – 9.3%), in order to produce a single measure of the multiple aspects of deprivation for each lower super output area (geographical area with approximately 1500 population).

Additional information on statistical methods

For analysis the cohorts were combined into four observation time periods – 2009 to 2011, 2012 to 2014, 2015 to 2017 and 2018 to 2019. 1^st^ January 2009 to 31^st^ December 2011 was treated as the reference period for all outcomes except kidney disease. Changes to the coding for hospital admission relating to dialysis in 2009 and 2010 meant that the number of recorded episodes of hospital care for kidney disease fell to artificially low levels and therefore the reference period for kidney disease was from 1^st^ January 2012 to 31^st^ December 2014, with follow up until 31^st^ December 2019. Follow up was limited to December 2019 to avoid the impact of the COVID-19 pandemic. No censoring occurred in the analysis of mortality but in the analysis of hospitalisations individuals were censored if they died during the follow up period. A table of the proportion of records censored by cause of hospitalisation is included in the Table S2.

The analyses of mortality were stratified into two age groups (20 to 74 years, and 75 years and older, in line with the UK government definition of premature mortality) and the analyses of hospitalisations were stratified into three age groups (20 to 49 years, 50 to 74 years, and 75 years and older); the event rate for mortality was too low in the 20 to 49 years age range to be analysed separately. To assess whether the associations between sex, ethnicity, socio-economic deprivation and duration of diagnosis differed between the age groups the confidence intervals for the RRs were compared. Non-overlapping confidence intervals for the RRs indicate a statistically significantly different association between that characteristic and mortality/hospitalisations between the age groups. To assess whether rates of change in mortality or hospitalisations varied by sex, ethnicity, socio-economic deprivation or by duration of diabetes, models with an interaction term of sex by time period, ethnicity by time period, quintile of deprivation by time period and duration of diabetes by time period were created. These analyses were stratified into two age categories (20 to 74 years, 75 years and older). The p-value reported in Supplemental Tables S5, S6, S7 and S8 indicates the statistical significance of the interaction term within the model (whether including the interaction term adds significantly to the explanatory power of the model). To assess whether the changes over time by the characteristics included in the interaction term (sex, socio-economic deprivation, ethnicity and duration of diagnosis) were significant, the confidence intervals for the RRs for the specific time period and the specific characteristic need to be considered. If the confidence intervals do not overlap then it is possible to say that the change over time is statistically significantly different based on the characteristic being analysed.

Whilst Poisson regression models are an appropriate and robust method to explore changes in hospitalisations and mortality over time, they are susceptible to over-dispersion which can lead to smaller p values and therefore a chance that results are incorrectly identified as statistically significant. In the main analyses the large cohort sizes mean that the majority of p values were very small (<0.001) and therefore the findings were unlikely to be altered. Nevertheless, the assumptions underlying Poisson regression models were assessed and over dispersion measured using Pearson’s chi-squared tests and the results were acceptable. Poisson regression models are non-collapsible which may result in the multi-variate adjusted co-efficients varying from univariate co-efficients and a lack of comparability between rate ratios in univariate and fully adjusted models. The co-efficients from the main fully adjusted models in this analysis align with univariate associations and with expected patterns. Despite these limitations, Poisson regression models provide a robust assessment of the changes over time explored in this analysis.

Explanation of the need for a 21 month period between end of the audit period and start of active follow-up in this analysis

The National Diabetes Audit (NDA) collates data on a time period from 1^st^ January in year one to 31^st^ March in year two (eg 1^st^ January 2016 to 31^st^ March 2017). There is a delay between the end of the audit time period and the date of data extraction from electronic systems. This means that people who die or transfer to another healthcare provider between the end of the audit period and the date of extraction are removed from the electronic system and details of their characteristics and care received during the audit period are not included in the NDA data extract. Over the time period of this analysis the length of the delay between the end of the audit period and data extraction has varied, and for a couple of audit periods, was very long. Any analysis of mortality and, to a lesser extent, complications, where the observation period for the events begins either straight after the end of the audit data collection period or in the time period between the end of the audit period and the date of the data collection taken with the longest delay from the end of the audit period will introduce bias due to the removal of a differing proportion of people from the data collection process across the years. This means that the only way to obtain consistent cohorts across the time period 2009 to 2019 is to apply a substantial (21 month) survival period after the end of the audit period.

Information Governance

NDA data is collected and used in line with NHS England’s purposes as required under the statutory duties outlined in the NHS Act 2006 and Health and Social Care Act 2012. There is controlled access by appropriately approved individuals, to data held on secure data environments entirely within the NHS England infrastructure. Data is processed for specific purposes only, including operational functions, service evaluations and service improvement. The data used to produce this analysis has been disseminated to NHS England under Directions issued under Section 254 of the Health and Social Care Act 2012. Ethics committee approval is not required for these specific purposes. Exclusion from the NDA is activated by an ‘opt-out’ system, the National Data Opt-out Service. The first author (NH) accessed the NDA data under an embedded researcher model involving an honorary contract with NHS England who collate and host the NDA. All numbers taken from the NDA are rounded to the nearest 5 to protect individuals’ confidentiality.
